# Proteomics Profiling Reveals the Molecular Signatures and Potential Therapeutic Targets of Human Nasopharyngeal Carcinoma

**DOI:** 10.1016/j.mcpro.2023.100567

**Published:** 2023-05-11

**Authors:** Ying Fu, Xujun Liang, Xinming Yang, Jianping Liu, Huichao Huang, Pengfei Zhang, Shisheng Li, Dandan Zhu, Ye Zhang, Fang Peng, Yongheng Chen, Zhuchu Chen

**Affiliations:** 1 Department of Oncology, NHC Key Laboratory of Cancer Proteomics & State Local Joint Engineering Laboratory for Anticancer Drugs, National Clinical Research Center for Geriatric Disorders, Xiangya Hospital, Central South University, Changsha, Hunan, China; 2 Department of Otolaryngology Head and Neck Surgery, The Second Xiangya Hospital, Central South University, Changsha, Hunan, China; 3 Institute for Advanced Study, Central South University, Changsha, Hunan, China

**Keywords:** nasopharyngeal carcinoma, proteomics, molecular signatures, therapeutic targets, nasopharyngeal carcinoma subtype

## Abstract

Nasopharyngeal carcinoma (NPC), a malignant tumor distinctly characterized by ethnic and geographic distribution, is highly prevalent in Southern China and Southeast Asia. However, the molecular mechanisms of NPC have not been fully revealed at the proteomic level. In this study, 30 primary NPC samples and 22 normal nasopharyngeal epithelial tissues were collected for proteomics analysis, and a relatively complete proteomics landscape of NPC was depicted for the first time. By combining differential expression analysis, differential co-expression analysis, and network analysis, potential biomarkers and therapeutic targets were identified. Some identified targets were verified by biological experiments. We found that 17-AAG, a specific inhibitor of the identified target heat shock protein 90 (HSP90), could be a potential therapeutic drug for NPC. Finally, consensus clustering identified two NPC subtypes with specific molecular features. The subtypes and the related molecules were verified by an independent data set and may have different progression-free survival. The results of this study provide a comprehensive understanding of the proteomics molecular signatures of NPC and provide new perspectives and inspiration for prognostic determination and treatment of NPC.

## Highlights

• A relatively complete proteomics landscape of NPC was depicted.
• The hallmark gene sets related to NPC and druggable target candidates were revealed.
• 17-AAG was demonstrated to be a potential therapeutic drug for NPC.
• Proteomics profiling identified two NPC subtypes with specific molecular features.

## Graphical Abstract

**Figure:**
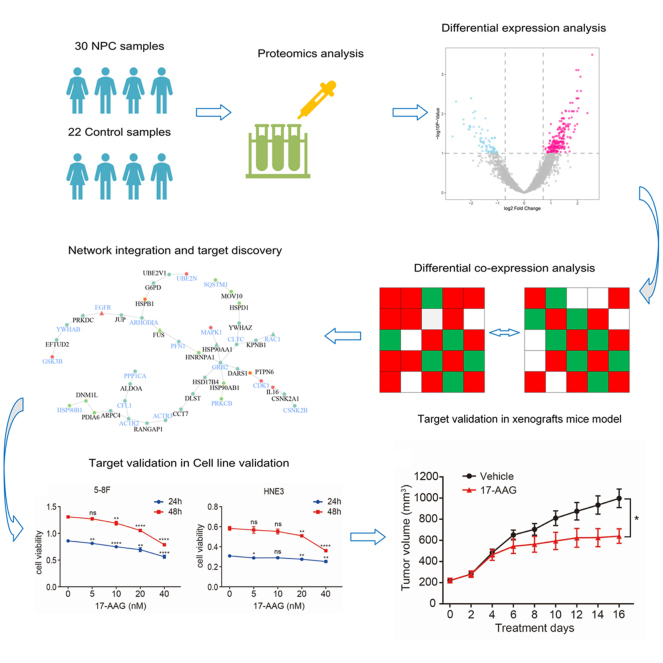

## In Brief

Proteomics analysis for 30 primary NPC and 22 control samples depicted a relatively complete proteomics landscape of NPC. By combining differential expression analysis, differential co-expression analysis, and network analysis, potential biomarkers and therapeutic targets were identified. HSP90 was identified as a potential target and its specific inhibitor 17-AAG was verified as a promising therapeutic drug for NPC *in vitro* and *in vivo*. In addition, consensus clustering identified two NPC subtypes with specific molecular features.

Nasopharyngeal carcinoma (NPC), a malignant tumor originating from nasopharyngeal epithelial cells, is distinctly characterized by ethnic and geographic distribution. It is highly prevalent in Southern China and Southeast Asia, with an annual incidence of 30∼50 per 100,000. The incidence in the United States and most other countries is under 5 per 100,000 (1). Globally, there is a consistent increase in NPC incidence and mortality. The number of new cases of NPC increased from 129,079 in 2018 to 133,354 in 2020. Meanwhile, the number of new cases of death increased from 72,987 in 2018 to 80,008 in 2020 (2, 3).

Currently, according to the World Health Organization (WHO) classification, NPC is classified into three major histopathological types based on the degree of differentiation. Type Ⅰ is keratinizing squamous cell carcinoma, which is also known to be well differentiated. Type II is moderately differentiated nonkeratinizing carcinoma, and type III is undifferentiated carcinoma. More than 95% of NPC patients in Southern China and Southeast Asia belong to type Ⅱ and type Ⅲ, but the prevalent pathological type in Western countries is type Ⅰ (4, 5). In addition to genetic susceptibility, Epstein–Barr virus (EBV) infection and environmental and dietary factors, such as consumption of preserved foods, are considered the main causative factors for NPC. At present, radiotherapy, especially intensity-modulated radiotherapy, is still believed to be the most effective and preferred approach in the treatment of NPC. In addition, the combination of cisplatin or fluorouracil-based chemotherapy is recommended for treating locally advanced tumors (1). Although great progress has been made in the management of NPC after decades of effort, the outcome is still unsatisfactory due to late presentation, distant metastasis, and local relapses.

The initiation and progression of NPC is a complex and consecutive process involving multiple molecules and multiple stages. Thus, in-depth exploration of the molecular alterations and pathogenesis underlying NPC can certainly help to improve diagnostic and treatment strategies in clinical practice. In recent years, with the advent and rapid development of high-throughput sequencing technologies, increasing genetic and epigenetic signatures of NPC have been uncovered (6). However, there have been no large-scale proteomics studies of NPC clinical tissue samples until now. Considering that proteins perform essential functions in the cell, an in-depth characterization of the NPC proteome will enable a more comprehensive understanding of the molecular mechanisms of NPC.

In this study, 30 primary NPC samples and 22 normal nasopharyngeal epithelial tissues from healthy people were collected for proteomics analysis. A total of 7243 protein groups were identified with a 1% false discovery rate (FDR) at the protein and peptide levels and depicted a relatively complete proteomics landscape of NPC. Gene Ontology enrichment analysis revealed that the upregulated proteins were significantly enriched in protein metabolism, apoptosis, and cell proliferation, while the downregulated proteins were significantly enriched in cell adhesion and lymphocyte activation. A total of 45,577 differentially co-expressed protein pairs and 500 proteins with differentially co-expressed links were identified by the R package diffcoexp. The most significantly differentially co-expressed link is glucose-6-phosphate isomerase (GPI), a known biomarker of cancer that is involved in glycolysis. Network-based integrative analysis of differential protein expression and differential co-expression revealed hallmark gene sets related to the inflammatory response and DNA repair in NPC. Additionally, the protein network suggested 15 druggable target candidates in NPC. Through cell viability and apoptosis detection in NPC cells, heat shock protein 90 (HSP90) was identified as a potential target, and its inhibitor 17-AAG was considered a possible therapeutic drug for NPC. Finally, by consensus clustering, we identified two NPC subtypes with specific molecular features that had different progression-free survival. Our integrative proteomics analysis made it possible to better comprehend the molecular signatures of NPC and may provide new insights and inspiration for the prognostic determination and treatment of NPC.

## Experimental Procedures

### Experimental Design and Statistical Rationale

The 30 NPC samples and 22 normal nasopharyngeal epithelial tissues samples used in this study were collected from the Second Xiangya Hospital of Central South University from March 2015 to December 2017. All the samples were obtained at the time of diagnosis upon their first visit and without undergoing any therapy. Every patients with NPC were diagnosed and confirmed by pathological examination according to the AJCC/UICC 7th Edition and the 2005 WHO classification. All the NPC samples used in this study were histopathologically diagnosed as nonkeratinizing carcinomas. Normal nasopharyngeal epithelial tissues were collected from patients diagnosed with chronic nasopharyngitis by nasal endoscopy during a biopsy. Detailed clinicopathologic information of the samples, including sex, age, and pathological diagnosis, was collected and shown in supplemental Table S1. The tissue samples were frozen in liquid nitrogen for storage immediately after collection. The statistical methods for analysis were described in the following sections.

### Ethics

The human studies reported in our manuscript abide by the Declaration of Helsinki principles. The study was approved by the Research Ethics Committee of Second Xiangya Hospital of Central South University. And written informed consent was obtained from all participants.

### Protein Extraction and Trypsin Digestion

The samples were washed with sterile phosphate-buffered saline (PBS) three times and lysed in 8 M urea supplemented with protease inhibitors and phosphatase inhibitors (Roche) for 30 min on ice. Then, the lysate was sonicated for 3 min (3 s on and 3 s off, amplitude 20%) and centrifuged at 12,000*g* for 15 min to remove the insoluble debris. The supernatant was transferred to a new EP tube, and the protein concentration was measured by BCA assay. Each aliquot of 500 μg total protein was reduced in 5 mM dithiothreitol at 56 °C for 30 min and then alkylated in 10 mM iodoacetamide at room temperature for 30 min in the dark. After dilution with 7 volumes of 50 mM ammonium bicarbonate and 1 M calcium chloride, the protein solution was digested with trypsin (enzyme to protein ratio at 1:50) at 37 °C for 16 h. An additional 0.1% formic acid (FA) was added to stop digestion, and the supernatant was moved into a new tube after centrifugation at 12,000*g* for 10 min.

### Peptide Fractionation and LC–MS/MS Analysis

The peptides were separated by using high-pH reversed-phase liquid chromatography as described previously (7). In brief, tryptic peptides were loaded and then fractionated on an XBridge BEH300 C18 column (Waters, 250 × 4.6 mm, OD 5 μm) at a flow rate of 0.7 ml/min. Mobile phase A [2% acetonitrile (ACN)-98% H_2_O, adjusted to pH 10.0 with NH_3_·H_2_O] and mobile phase B (98% ACN-2% H_2_O, adjusted to pH 10.0 with NH_3_·H_2_O) were used for peptide elution. A 72 min gradient separation was set as follows: 5%–8% B in 5 min; 8%–18% B in 35 min; 18%–32% B in 22 min; 32%–95% B in 2 min; 95% B for 4 min; and 95%–5% B in 4 min. The eluate was collected every 1 min and combined into six fractions before freeze-drying by vacuum centrifugation.

The fractionated peptides were analyzed on an Easy-nLC 1000 LC system (Thermo Fisher) coupled with Orbitrap Fusion mass spectrometry (Thermo Fisher) and a nanoelectrospray ion source (Thermo Fisher). Briefly, peptides redissolved in buffer A (0.1% FA) were directly loaded onto a 2 cm long, homemade trap column (100 μm inner diameter, particle size 3 μm, pore size 100 A°; SunChrom) and separated on a 15 cm long C18 nanocapillary analytical column (150 μm inner diameter, particle size 3 μm, pore size 100 A°; SunChrom) with a 120 min gradient (buffer A, 0.1% FA in water; buffer B, 0.1% FA in ACN) at a flow rate of 350 nl/min. The gradient was set as follows: 0 to 5 min, 5% buffer B; 6 to 80 min, 5% to 20% buffer B; 81 to 98 min, 20% to 30% buffer B; 99 to 104 min, 30% to 95% buffer B; and 105 to 120 min, 95% to 5% buffer B. The fractioned peptides were ionized under 2 kV, and one full mass spectrometry scan was operated under an up to 20 data-dependent acquisition mode by high-energy collision dissociation (normalized collision energy of 35%). The MS1 full scan was set at a resolution of 60,000, and the ions with m/z ranged from 280 to 1500. The MS2 spectrum was acquired in top-speed mode with an AGC target of 7000. The maximal time for ion injection was 35 ms, and the maximal time for dynamic exclusion was 60 s.

### Database Search for Protein Identification and Quantification

All raw mass spectrometric files were searched against the human UniProt database (8) (downloaded in May 2017, containing 20,198 sequences) by using MaxQuant (9) (version 1.5.3.17). The protease was set as Trypsin/P. The maximum number of missed cleavages was set as 2. Carbamidomethyl (C) was included as a fixed modification. In protein modification, oxidation of M and protein N-terminal acetylation were included. The FDR of both the proteins and peptides was set to 0.01. The detail information of the identified proteins was presented in supplemental Table S2. Only proteins with at least two unique peptides were retained for the following analysis. The intensity-based absolute quantification (iBAQ) method was utilized to calculate the relative protein abundance. The iBAQ values in the MaxQuant results were log2 transformed and normalized so that the iBAQ values of each sample had the same quantiles. To increase the reliability of the analysis, only proteins with less than 40% missing data in all samples were retained. Then, the remaining missing values were imputed by the K-nearest neighbor method using the R package *impute* (10). The distributions of iBAQ values of proteins in each sample were visualized in supplemental Fig. S1.

### Bioinformatics Analysis

#### Differential Protein Expression Analysis

After data filtering and imputing as described above, proteomic data without missing values (4121 proteins in total) were used to identify proteins differentially expressed between 22 control samples and 30 nasopharyngeal carcinoma samples. The linear model implemented in the R package *limma* was used to calculate the statistical significance of protein differential expression (11).

#### Differential Co-Expression Analysis of Proteins

The R package *diffcoexp* (12) was utilized to find the differential co-expression patterns of proteins. First, the Pearson correlations of each pair of proteins in NPC and control samples were calculated separately. Then, the differences in the correlation coefficients between the NPC and control groups were evaluated, and the statistical significance of the differences was estimated by Fisher's Z-transformation. A protein pair that was defined as a differential co-expression link satisfied the following criteria: (1) the correlation coefficient was larger than 0.5 and FDR<0.1 in either type of sample; (2) the difference in correlation coefficients was larger than 0.5 and FDR<0.1. Furthermore, proteins were identified as differentially co-expressed proteins if they had more differentially co-expressed links than expected (FDR<0.1).

#### Network Construction and Analysis

Protein–protein interaction (PPI) data were downloaded from the HIPPIE database (13). The self-interactions of proteins were removed from the PPI network. Then, the PPI network and the adjusted differential expression *p* values obtained by *limma* were used as the input for the R package *LEANR* (14). *LEANR* could identify subnetworks enriched for differentially expressed proteins. The subnetworks were further filtered to keep only those enriched for differential co-expression links. The protein links that were not significantly differentially co-expressed were removed from these subnetworks. The maximum connected components of the remaining subnetworks were merged to obtain a unified network. The R package *PCSF* was used to extract the subnetwork related to a special gene set (15).

#### Gene Ontology and Pathway Analysis

Gene ontology enrichment and hallmark gene set enrichment analyses were performed with the R package *clusterProfile* (16) and visualized with ClueGo (17). Pathway enrichment analysis for differential co-expression links was performed by using Fisher’s exact test, and the *p* values were adjusted *via* Benjamini–Hochberg’s method.

#### Consensus Clustering of NPC Samples

The filtered and imputed protein expression data were used for clustering with the R package *ConsensusClusterPlus* (18). The number of subsamples was set to 1000, and the maximum cluster number was set to 6. All the other parameters were set as default. Here, the consensus matrix of k = 2 gave clear separation clusters and a balanced number of samples in each cluster. Therefore, the number of clusters was set to 2.

### Cell Culture and Cell Viability Assay

Human NPC cell lines 5-8F and HNE3 were cultured in RPMI-1640 medium supplement with 10% fetal bovine serum and 1% penicillin/streptomycin at 37 °C in a humidified 5% CO_2_ atmosphere. Cell cytotoxicity and viability assays were performed by using CCK-8 (Dojindo) according to the manufacturer’s instructions. The chemicals 17-AAG, NMS-873, and Ro-3306 were purchased from Selleck (Selleck Chemicals) and dissolved in dimethyl sulfoxide (DMSO). For IC50 determination, 5-8F and HNE3 cells were seeded into 96-well plates at 2 × 10^3^ cells per well and treated with a series dilution of the three chemicals mentioned above for another 72 h. Cell viability was examined by a PowerWave XS Microplate Reader (BioTek) at 450 nm absorbance after incubation with CCK-8 for 1 h. The values were determined using GraphPad Prism 7.

### Colony Formation Assay

Cells were seeded in 6-well plates at a density of 500 per well and treated with DMSO or 17-AAG (10, 20, and 40 nM). After culturing for 10 days, the colonies were fixed with 4% paraformaldehyde and stained with 0.1% crystal violet for 30 min. After washing, the stained colonies were photographed and counted. The experiment was carried out in triplicate for each cell line.

### Flow Cytometric Analysis of Apoptosis

Cells were plated in 6-well plates and treated with DMSO or 17-AAG (10, 20, and 40 nM) for 48 h. After digestion with trypsin, the cells were collected and resuspended in 500 μl of 1 × binding buffer. Then, 5 μl Annexin V-FITC and 5 μl PI were added to the cells and incubated for 15 min in the dark at room temperature. The cells were analyzed by flow cytometry (BD Biosciences).

### Western Blotting

After treatment with DMSO or 17-AAG (5, 10, 20, 40 nM) for 48 h, the cells were lysed in RIPA lysis buffer. An equal amount of protein in each sample was subjected to SDS–PAGE separation and transferred to PVDF membranes. After blocking with 5% nonfat milk, the membranes were incubated with primary antibodies caspase-3 (Proteintech Cat# 19677-1-AP), poly (ADP-ribose) polymerase (PARP) (Cell Signaling Technology Cat# 9542), and β-tubulin (Proteintech Cat# 10094-1-AP) overnight at 4 °C, followed by incubation with horseradish peroxidase–conjugated secondary antibody for 1 h at room temperature. The bands were visualized *via* chemiluminescence.

### Tumor Xenograft Experiment

A total of 5 × 10^6^ HNE3 cells in PBS mixed with Matrigel (1:1) were subcutaneously injected into the right flanks of four-week-old BALB/c mice to generate xenografts. When the mean tumor volume reached approximately 200 mm^3^, the mice were divided into two groups and were intraperitoneally injected with vehicle (90% corn oil, 10% DMSO) or 17-AAG (40 mg/kg) every other day, and the body weights were recorded. The tumor size was measured using a caliper every other day, and the tumor volume was calculated with the formula V = π/6 × length (mm) × width (mm) × height (mm). After 16 days of treatment, the mice were sacrificed, and the tumors were collected and weighted. All daily animal care and the experimental procedures were approved by the Animal Ethics Committee of Central South University.

### Statistical Analysis

Statistical analyses were performed using SPSS 23.0 and GraphPad Prism 7 software. Unpaired Student’s *t* test or one-way ANOVA was used for comparisons between groups, and the data are expressed as the mean ± SD. *p* < 0.05 was considered statistically significant. The survival analysis in this work was carried out by using the Kaplan–Meier method, and statistical significance was obtained by the log-rank test. The R package *survival* was used to implement the analysis (19).

## Results

### Proteomics Profile of Nasopharyngeal Carcinoma

**Fig. 1:**
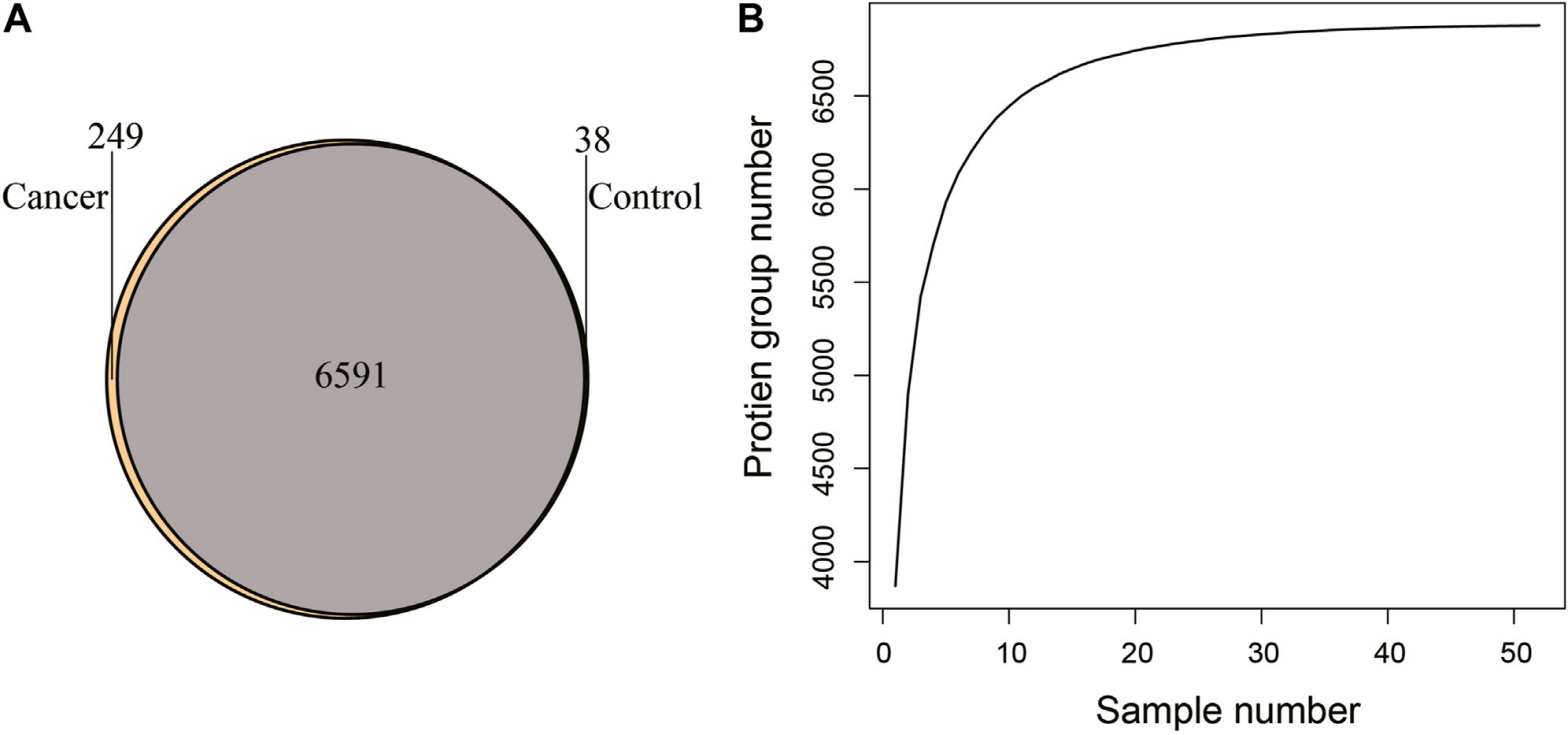
**An overview of the proteomics profile of NPC.***A*, Venn diagram showing the common protein groups and the specific protein groups identified in NPC samples and control samples. *B*, cumulative curve for the number of identified proteins with respect to the number of samples. NPC, nasopharyngeal carcinoma.

In this study, 30 primary NPC samples and 22 control samples from healthy people were collected for proteomics analysis. All raw files produced by MS runs were analyzed together, and 7243 protein groups were identified with a 1% FDR at the protein and peptide levels. Among these protein groups, 6591 (90.1%) protein groups were present in both NPC and control samples, 249 protein groups were only present in NPC samples, and 38 protein groups were uniquely detected in control samples (Fig. 1*A*). It was also observed that the number of protein groups identified by MS did not increase dramatically after 25 samples were examined (Fig. 1*B*). Supplemental Fig. S2 shows that the identified proteins were distributed throughout the majority of cells. These results suggest that the analysis of this study could provide a relatively complete proteomic landscape of NPC.

### Differential Protein Expression Between NPC and Control Samples

**Fig. 2:**
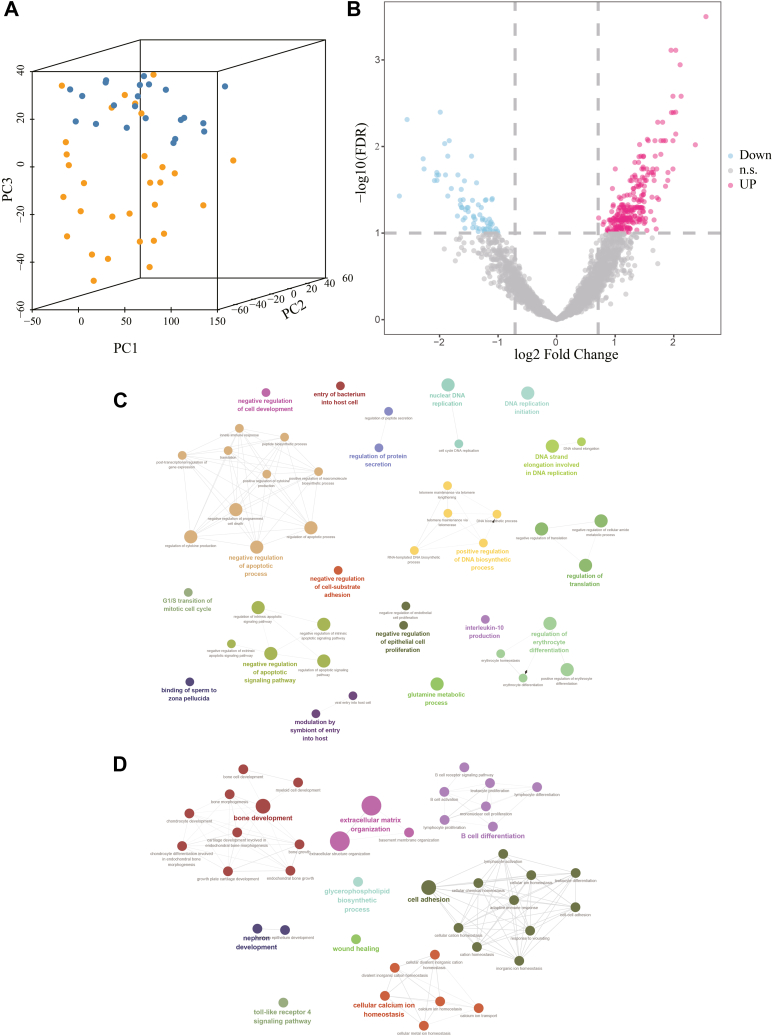
**Differentially expressed proteins between NPC samples and control samples.***A*, PCA analysis is visualized with the three largest principal components. *Bule points* denote the control samples, and *orange points* denote the NPC samples. *B*, volcano plot for the differential protein expression. *Red points* denote the upregulated protein groups, while *blue points* denote the downregulated protein groups. n.s. means not significant. *C*, top-ranked biological processes that were significantly enriched by the upregulated proteins. *D*, top-ranked biological processes that were significantly enriched by the downregulated proteins. NPC, nasopharyngeal carcinoma; PCA, principal components analysis.

After filtering, imputing, and normalizing (see the Methods section for details), the iBAQ values of 4121 proteins were used to find the proteomic differences between NPC and control samples. In principal components analysis, the first three principal components were extracted to visualize the overall proteomic differences between NPC and control samples. Figure 2*A* shows that most NPC samples could be separated from control samples, especially at the second and third principal components (PC2 and PC3). This result indicates that the proteomic profile is changed in NPC. Using the linear model with empirical Bayes smoothing to the standard errors in the R package *limma* (11), a total of 317 protein groups were identified to be differentially expressed between NPC and control samples (FDR<0.1 and ratio of NPC over control or vice versa >1.5). There were 244 upregulated proteins and 73 downregulated proteins (Fig. 2*B* and supplemental Table S3). Gene Ontology enrichment analysis revealed that the upregulated proteins were significantly enriched in DNA biosynthetic process, apoptosis, and cell proliferation (FDR<0.1, Fig. 2*C*), while the downregulated proteins were significantly enriched in cell adhesion and lymphocyte activation (FDR<0.1, Fig. 2*D*).

### Differential Co-Expression of Protein Pairs Between NPC and Control Samples

In addition to identifying differentially expressed proteins, differential protein co-expression patterns were also explored in this study. Differential co-expression analysis is aimed at detecting the altered co-expression patterns of protein pairs between two classes of samples. These altered protein co-expression patterns may represent direct protein interactions or indirect protein associations affected by disease status (20). Here, we identified the differentially co-expressed protein pairs with the R package *diffcoexp* (12). According to the criteria described in the Methods section, 45,577 differentially co-expressed protein pairs were found (Supplemental Table S4). Among these differential co-expression links, 982 protein pairs had records of direct interactions in the HIPPIE database (13) or causal relationships in the SIGNOR database (21). Some of these protein pairs have been reported to play important roles in cancer. For example, SRC can enhance the phosphorylation of epidermal growth factor receptor (EGFR) and promote cell transformation and cancer development (22). In our results, EGFR and SRC had no significant correlation in the control samples (correlation coefficient = −0.19, FDR = 0.72), while they showed a strong positive correlation in the NPC samples (correlation coefficient = 0.70, FDR = 3.9e-3). Protein tyrosine phosphatase nonreceptor type 6 (PTPN6) can dephosphorylate signal transducer and activator of transcription 3 (STAT3) and suppress its activity (23). There was also some evidence that STAT3 activation contributed to the development of NPC (24). In our results, PTPN6 and STAT3 were positively correlated in the control samples (correlation coefficient = 0.84, FDR = 5.3e-4), but they were not significantly correlated in the NPC samples (correlation coefficient = −0.24, FDR = 0.60). This finding suggests that the regulation of STAT3 activity by PTPN6 may tend to weaken in NPC.

To further illustrate the biological significance of the differentially co-expressed protein pairs, we examined the Kyoto Encyclopedia of Genes and Genomes (KEGG) pathways to which these protein pairs were related. As shown in Table 1, there were 25 significantly enriched KEGG pathways (FDR<0.1). The pentose phosphate pathway and glycolysis/gluconeogenesis pathway were the most significant pathways enriched with differentially co-expressed protein pairs. The pentose phosphate pathway has been reported to be crucial for the cellular metabolism of cancer cells (25, 26). A previous study showed that the glycolytic pathway may regulate the cancer stem cell properties of NPC (27). The differentially co-expressed protein pairs were also related to some other important pathways associated with cancer, such as the cell cycle and MAPK signaling pathway.

**Table 1:** KEGG pathways enriched by significantly differentially co-expressed protein links

Pathway	*p* value	FDR	Differential co-expression protein pairs in pathway
Pentose phosphate pathway	3.80E-14	3.84E-12	PGLS-GPI;GPI-PGM2;G6PD-PGM1;GPI-PGM1;PGLS-TALDO1;PGM1-PGD;PFKL-TKT;PFKL-TALDO1;PGD-PRPS1;G6PD-PFKL
Glycolysis gluconeogenesis	3.26E-12	1.65E-10	GPI-DLAT;GPI-PDHB;GPI-PDHA1;GPI-PGM2;GPI-ADH5;GPI-DLD;PGK1-GPI;ENO1-GPI;GPI-PGM1;ENO1-PGM1
Spliceosome	4.99E-10	1.68E-08	HSPA1B-LSM3;HSPA1A-LSM3;HSPA1A-LSM3;HSPA1B-LSM3;SNRPB2-NCBP1;SRSF6-DHX38;HSPA1A-PRPF8;HSPA1B-PRPF8;HSPA1A-PRPF8;HSPA1B-PRPF8
MAPK signaling pathway	1.15E-09	2.90E-08	HSPA1B-GRB2;HSPA1A-GRB2;HSPA1A-GRB2;HSPA1B-GRB2;PRKACA-DUSP3;HSPA1A-PAK2;HSPA1B-PAK2;HSPA1A-PAK2;HSPA1B-PAK2;HSPA8-STK4
Pathways in cancer	2.53E-08	5.11E-07	KRAS-HSP90AB1;HSP90AA1-GRB2;KRAS-HSP90AA1;GSTP1-RAC2;GSTP1-GRB2;STAT3-CYCS;HSP90AB1-STK4;KRAS-GSTP1;HSP90AB1-RAC2;RALB-TFG
Proteasome	6.93E-07	1.17E-05	PSMC2-PSMD7;PSMD7-PSMC6;PSMD14-PSMC1;PSMC1-PSME2;PSMA7-PSMC1;PSMD12-PSMC1;PSMB9-PSMD7;PSMD7-PSMC1;PSME2-PSMD13;PSMB8-PSMC6
Pathogenic *Escherichia coli* infection	3.32E-06	4.79E-05	ARPC2-NCL;RHOA-TUBB4B;ARPC1B-NCL;TUBB-ARPC4;ARPC3-NCL;ARPC3-TUBA1C;WAS-CTTN;ARPC1B-ARPC1A;ARPC5-TUBB4B;NCL-RHOA
Prostate cancer	7.55E-06	9.53E-05	KRAS-HSP90AB1;HSP90AA1-GRB2;KRAS-HSP90AA1;GSTP1-GRB2;KRAS-GSTP1;HSP90AB1-NFKB1;HSP90AB1-MAPK1;HSP90AB1-GRB2;HSP90AA1-MAPK1;HSP90B1-GRB2
Endocytosis	2.32E-05	0.00026	GRK6-SNF8;HSPA8-DNM2;RAB5C-PDCD6IP;HSPA1B-EHD1;HSPA1A-EHD1;HSPA1B-EHD1;HSPA1A-EHD1;TFRC-SH3GLB1;HSPA8-RAB5C;ACAP2-TSG101
Progesterone-mediated oocyte maturation	2.81E-05	0.000284	GNAI2-HSP90AB1;KRAS-HSP90AB1;CDK1-RPS6KA3;KRAS-HSP90AA1;CDK1-MAPK3;HSP90AB1-MAPK1;GNAI2-CDK1;HSP90AA1-MAPK1
Vasopressin-regulated water reabsorption	6.63E-05	0.000609	ARHGDIA-DYNC1H1;RAB5C-DYNC1H1;RAB5C-GNAS;RAB5C-GNAS;NSF-RAB5C;NSF-RAB11 B;DYNLL1-DYNC1H1;ARHGDIB-DYNC1H1;NSF-DYNC1H1
Glyoxylate and dicarboxylate metabolism	0.000176	0.001482	MTHFD1-ACO1;CS-MTHFD1;MTHFD1-MDH1;MTHFD1-ACO2
Nod-like receptor signaling pathway	0.00041	0.003185	HSP90AB1-NFKB1;HSP90AB1-MAPK1;HSP90AA1-MAPK1;HSP90AB1-PYCARD;HSP90AA1-HSP90B1;CASP8-PYCARD
Adherens junction	0.001414	0.010202	RAC2-CSNK2A1;RHOA-CSNK2A1;IQGAP1-CSNK2A1;PTPN6-CSNK2A1;CSNK2B-CSNK2A1;WAS-CSNK2A1;CTNND1-RHOA;SRC-RAC2;MAPK1-RAC3;MAPK1-RAC1
Fc gamma R-mediated phagocytosis	0.00159	0.010706	DNM1L-RAC1;DNM2-ARPC1A;ARPC1B-ARPC1A;GSN-ARPC5L;DNM1L-ARPC5L;DNM1L-PLCG2;MAPK1-RAC1;RAC1-ARPC1A;RAC2-CRKL;ARPC1B-CRKL
Amyotrophic lateral sclerosis ALS	0.002746	0.017331	CAT-CYCS;CAT-RAC1;CAT-GPX1;CAT-PPP3CA;PPP3CB-BAX
Glutathione metabolism	0.004437	0.024938	GSTA1-GSTP1;TXNDC12-PGD;SRM-PGD;G6PD-SRM;GPX1-G6PD;GSTA1-PGD;GSR-G6PD
Cell cycle	0.004444	0.024938	YWHAB-PRKDC;YWHAB-SMC3;PCNA-YWHAB;HDAC1-SMC1A;PRKDC-HDAC1;GSK3B-HDAC1;CDC27-HDAC2;CDC27-PRKDC;CDC27-HDAC1;CDC27-SMC1A
Amino sugar and nucleotide sugar metabolism	0.00667	0.034052	GMDS-GPI;GPI-PGM2;GPI-UGP2;GPI-GMPPA;GPI-PGM1;GPI-NAGK;TSTA3-GMPPB
Starch and sucrose metabolism	0.006743	0.034052	GPI-PGM2;GPI-UGP2;GPI-PGM1;PYGB-HK1
Chemokine signaling pathway	0.008474	0.040754	CSK-STAT2;STAT2-GNB1;STAT3-CSK;GNAI2-STAT1;RAC2-STAT2;RAC2-STAT3;STAT2-RHOA;KRAS-STAT3;STAT3-GRB2;GNAI2-STAT2
Prion diseases	0.010362	0.047572	HSPA1A-MAPK1;HSPA1A-MAPK1;HSPA1A-STIP1;HSPA1A-STIP1
Leukocyte transendothelial migration	0.013534	0.059433	CTNND1-RHOA;CYBA-CTNNA1;ITGAM-CTNNA1;ACTN4-EZR;CTNND1-ITGAM;MYL12A-ITGAL;MYL12B-ITGAL;MYL12A-ITGAL;ROCK2-GNAI2;ROCK2-CDC42
Drug metabolism cytochrome P450	0.019058	0.080201	GSTA1-GSTP1;GSTP1-ADH5;GSTM3-MAOA
JAK STAT signaling pathway	0.02211	0.089326	PTPN6-STAT2;PTPN6-STAT3;PTPN6-STAT1;STAT3-GRB2

**Fig. 3:**
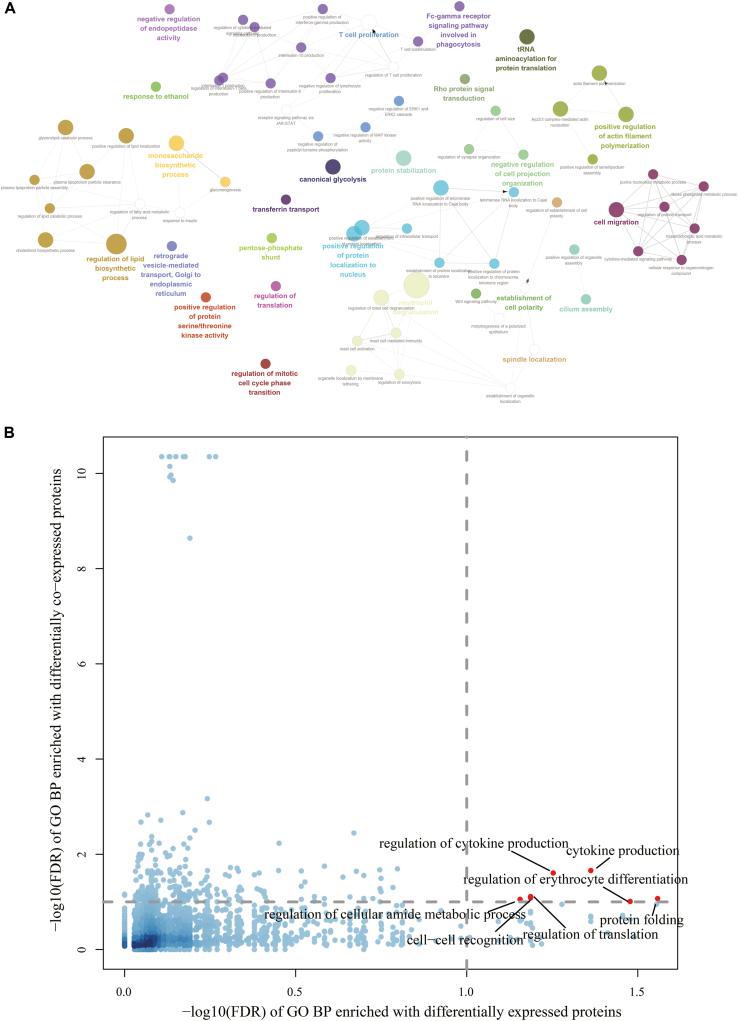
**Biological processes enriched with proteins that have significantly more differentially co-expressed links.***A*, the top-ranked biological processes. *B*, comparison of enriched biological processes between differentially expressed proteins and differentially co-expressed proteins. The common significantly enriched biological processes are shown as *red points*. FDR, false discovery rate.

In addition to differentially co-expressed protein pairs, the enrichment of differential co-expression links of each protein was also tested with *diffcoexp*. It was found that 500 proteins had significantly more differentially co-expressed links (FDR <0.1, Supplemental Table S5), and 25.5% of these proteins were also differentially expressed between the control and NPC samples. The protein with the most significantly differentially co-expressed links is encoded by the *GPI* gene. This protein was also highly expressed in the NPC samples. GPI is involved in glycolysis and has cytokine activity (28, 29). It is a known biomarker of cancer (30). We also observed that GPI was significantly associated with progression-free survival (*p* value = 0.02, log-rank test) by analyzing the mRNA expression data from a previous study (31). The other two top-ranked proteins encoded by the cytoplasmic linker associated protein 1 gene and the cell division cycle 27 are related to the cell cycle. Gene otology enrichment analysis showed that these differentially co-expressed proteins participate in some immune-related biological processes (Fig. 3*A*). Interestingly, the enriched biological processes of these proteins dramatically differed from the differentially expressed proteins, and only seven biological process terms were significantly enriched in both of the enrichment analyses (Fig. 3*B*). This implies that the differential co-expression analysis is complementary to the differential expression analysis.

### Network-Based Integrative Analysis of Differential Protein Expression and Differential Co-Expression

To further explore the underlying molecular mechanism of NPC, we integrated the protein differential expression and differential co-expression results with a network-based analysis framework. First, the differentially expressed proteins were mapped to the PPI network retrieved from the HIPPIE database. Next, the R package *LEANR* was employed to identify the dysregulated local subnetworks consisting of a center protein and the proteins directly interacting with it in a given network (14). A total of 318 significant local subnetworks were obtained (FDR <0.1). Then, we examined whether the proteins in these subnetworks were also differentially co-expressed. It was found that 158 out of these 318 subnetworks were significantly enriched with differentially co-expressed protein links (Fisher’s exact test, adjusted *p* value < 0.1 by Holm method, Supplemental Table S6). These 158 subnetworks were retained for the following analysis.

**Fig. 4:**
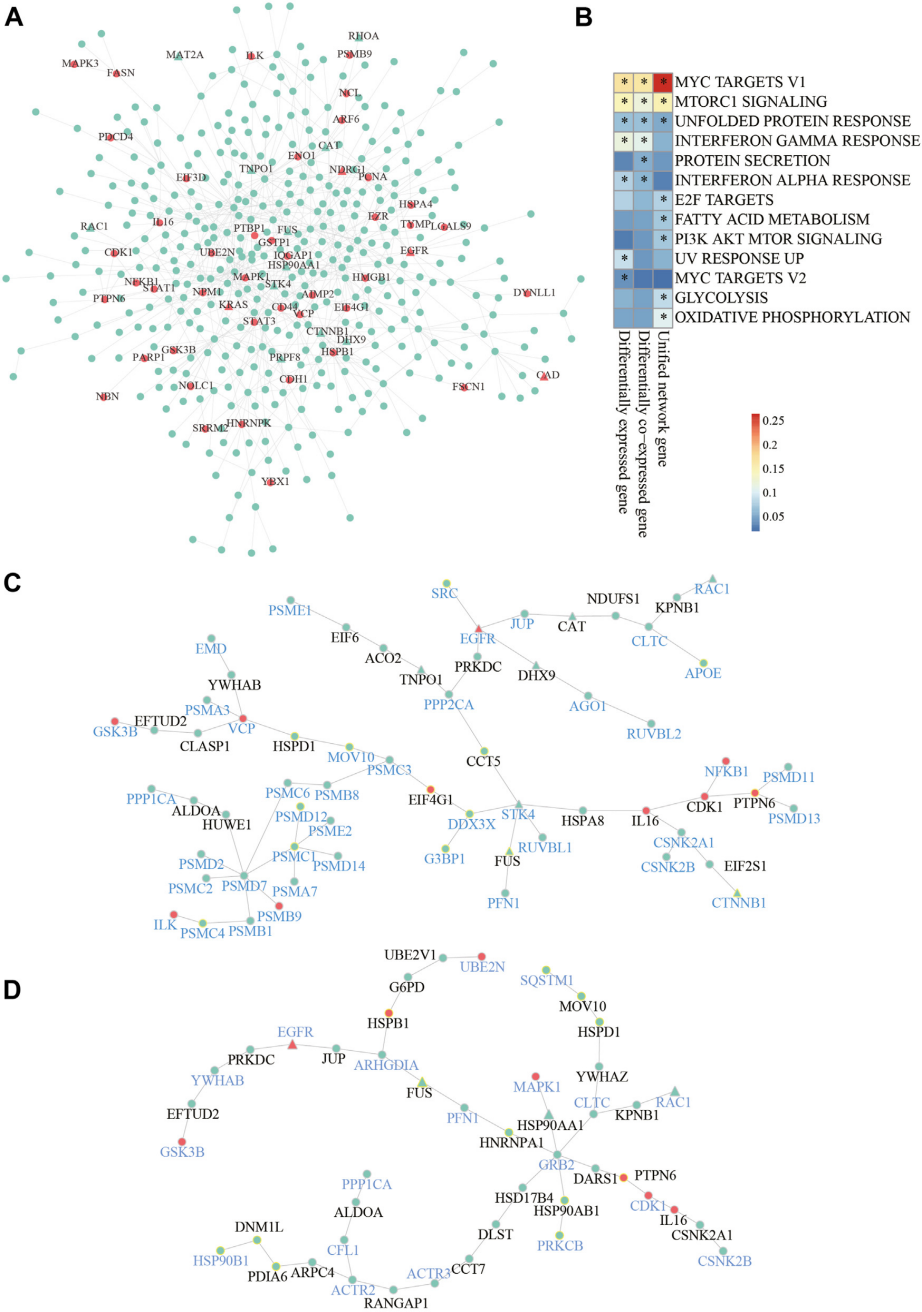
**Integrative analysis of protein differential expression and differential co-expression.***A*, the molecular network for NPC. *B*, cancer-related hallmark gene set enrichment analysis. The *star symbols* denote the significantly enriched gene sets. *C*, Wnt signaling pathway-related subnetwork. *D*, PI3K-AKT-MTOR pathway-related subnetwork. For networks in *A*, *C*, and *D*, *red nodes* are NPC-related genes from DisGeNET, and *triangle nodes* are candidate cancer drug targets. In *C* and *D*, nodes with *yellow edges* are genes in the Wnt signaling pathway or PI3K-AKT-MTOR pathway. The other nodes are link nodes from the network in *A*. NPC, nasopharyngeal carcinoma.

**Fig. 5:**
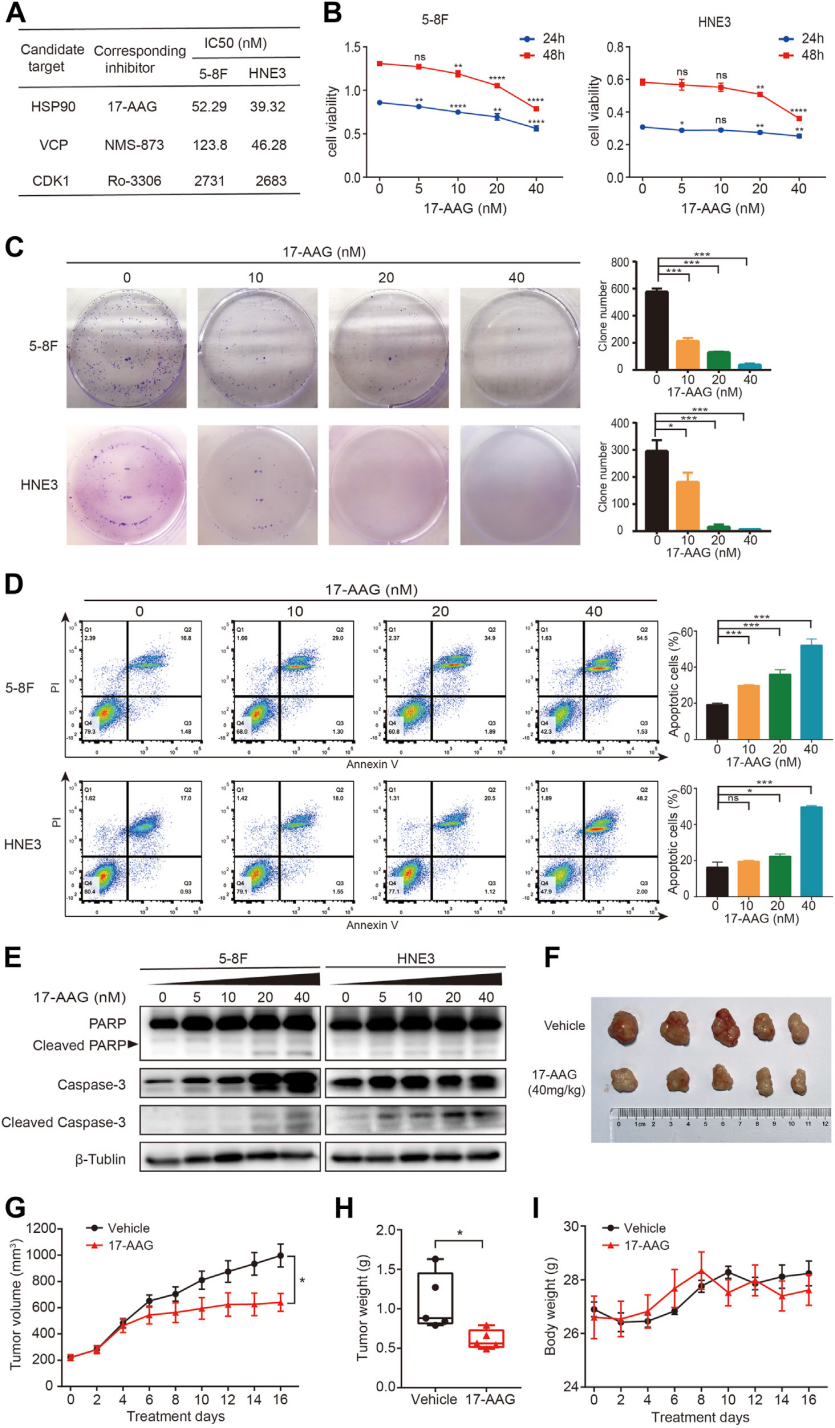
**Evaluation of the potential drug targets in NPC.***A*, the IC50 of the corresponding inhibitors for candidate targets in 5-8F and HNE3 cells. *B*, the viability of 5-8F and HNE3 cells upon treatment with different doses of 17-AAG for 24 h or 48 h. *C*, the effects of different doses of 17-AAG on the colony formation of 5-8F and HNE3 cells. *D*, the apoptosis of NPC cells induced by different concentrations of 17-AAG was detected by flow cytometry using Annexin V/PI staining, and statistical analysis of the apoptotic cell percentage is shown in the right panel. *E*, Western blotting analysis of the effects of 17-AAG on PARP, cleaved PARP, caspase-3, and cleaved caspase-3 in NPC cells. *F*, images of excised tumors after treatment with 17-AAG or vehicle in BALB/c mice (n = 5). *G*, the change of tumor volume in subcutaneous models was shown. *H*, the weight of subcutaneous xenografts tumors. *I*, 17-AAG treatment had no significant effects on the body weight of mice. The data are presented as the mean ± SD from three independent replicates. ns indicates not significant, ∗*p* < 0.05, ∗∗*p* < 0.01, ∗∗∗*p*< 0.001. CDK1, cyclin-dependent kinase 1; HSP90, heat shock protein 90; NPC, nasopharyngeal carcinoma; PARP, poly (ADP-ribose) polymerase; VCP, valosin-containing protein.

To further integrate the subnetworks identified with differentially expressed proteins and the results of differential co-expression analysis, we removed the protein links in the subnetworks that were not significantly co-expressed and kept only the maximum connected component of each subnetwork. It was noticed that these subnetworks had some overlapping nodes and edges. For example, the two subnetworks that were most significantly enriched with differential co-expression links are shown in supplemental Fig. S3. These two subnetworks had 84 common nodes and 94 common edges. Accordingly, the proteins in these two subnetworks participated in some common biological processes, such as DNA metabolic processes, translation, and telomere maintenance. Because the subnetworks overlapped, we constructed a unified molecular network for NPC containing 449 proteins and 682 links by combining all subnetworks (Fig. 4*A*). The unified network revealed some biological processes that were not enriched in the differential expression analysis or the differential co-expression analysis (the enrichment results can be found in Supplemental Table S7). For example, the Wnt signaling pathway was enriched only in the unified network, and the Wnt pathway is known to play an important role in NPC development (32). It was also noticed that 46 NPC-related genes recorded in the DisGeNET database (33) were located in the unified network (Fig. 4*A*), which confirmed the validity of our analysis. In addition, the unified network was significantly represented by several cancer-related hallmark gene sets from the MSigDB database (34). As shown in Figures 4*B* and 5 hallmark gene sets were only enriched in the unified network. However, there were also some hallmark gene sets related to the inflammatory response and DNA repair that were specifically enriched in the differentially expressed genes or differentially co-expressed genes. These results imply that different analyses provide complementary views to understand the proteomics landscape of NPC. Among the enriched hallmark gene sets, IFN-gamma response could be inhibited by EBV (35), while EBV is closely associated with NPC (36). Here, we leveraged the R package *GSVA* (37) to estimate the activity of EBV infection pathway defined by the KEGG database (hsa05169) as well as the activity of IFN-gamma response in each NPC sample. It was found that the activity of EBV infection pathway was positively correlated with the activity of IFN-gamma response (Pearson's correlation, R = 0.54, *p*-value = 0.002, supplemental Fig. S4).

Additionally, the unified protein network suggested some therapeutic candidates. We retrieved 159 druggable cancer target candidates from a previous study (38) and found that 15 of these targets were in the unified network (Fig. 4*A*). Some of these targets may affect important NPC pathways. For example, EGFR is usually aberrantly expressed in NPC, and anti-EGFR-targeted therapeutic agents show promise in NPC clinical practice (39). Another enriched protein, encoded by the valosin-containing protein (also known as *p97*), was in the center node of the Wnt signaling pathway subnetwork and was demonstrated to be overexpressed in many cancer types and is regarded as a potential biomarker and therapeutic target (40) (Fig. 4*C*). PI3K-AKT-MTOR signaling was enriched in the unified network. The PI3K signaling pathway was reported to be significantly mutated in NPC (41). There were several druggable targets in the PI3K-AKT-MTOR subnetwork extracted from the unified network (Fig. 4*D*). Growth factor receptor bound protein 2 (GRB2) was in the center of this subnetwork. Although GRB2 is not in the list of druggable candidates, some studies have shown that GRB2 is a promising cancer therapeutic target (42, 43).

### Identification of Potential Drug Targets in NPC

From the unified protein network, we chose HSP90, valosin-containing protein, and cyclin-dependent kinase 1 (CDK1), which are enriched in the nodes of the signaling pathways and tried to validate their potential as drug targets in NPC. First, the corresponding inhibitors of candidate targets were used to examine the IC50 in NPC cell lines 5-8F and HNE3. The IC50 values of the three inhibitors are shown in Figure 5*A* and 17-AAG, which is an effective inhibitor of HSP90, exhibited the strongest inhibitory activity in NPC cells. Subsequently, CCK8 and colony formation assays were performed to evaluate the effects of 17-AAG on the viability and proliferation of NPC cells. The results showed that 20 nM 17-AAG significantly suppressed the viability and proliferation of NPC cells (Fig. 5, *B* and *C*). Annexin V/PI staining in combination with flow cytometry analysis revealed that 17-AAG obviously promoted NPC cell apoptosis (Fig. 5*D*). In addition, Western blotting was carried out to detect the expression of PARP, cleaved PARP, caspase-3, and cleaved caspase-3 in 5-8F and HNE3 cells after treatment with different concentrations of 17-AAG. As shown in Figure 5*E*, the expression of these apoptosis-related proteins in NPC cells increased after treatment with 17-AAG in a concentration-dependent manner. To further determined the antitumor efficacy of 17-AAG *in vivo*, we established HNE3 xenografts mice model and intraperitoneal injected with 40 mg/kg 17-AAG every other day. The results revealed that 17-AAG treatment can obviously reduce tumor volume and weight in xenografts mice compared to control group, with no obvious change in body weight (Fig. 5, *F–I*). All of these results indicated that HSP90 might be a potential drug target, while its inhibitor 17-AAG could be a potential therapeutic drug for NPC.

### NPC Proteomics Subtypes and Molecular Characteristics

**Fig. 6:**
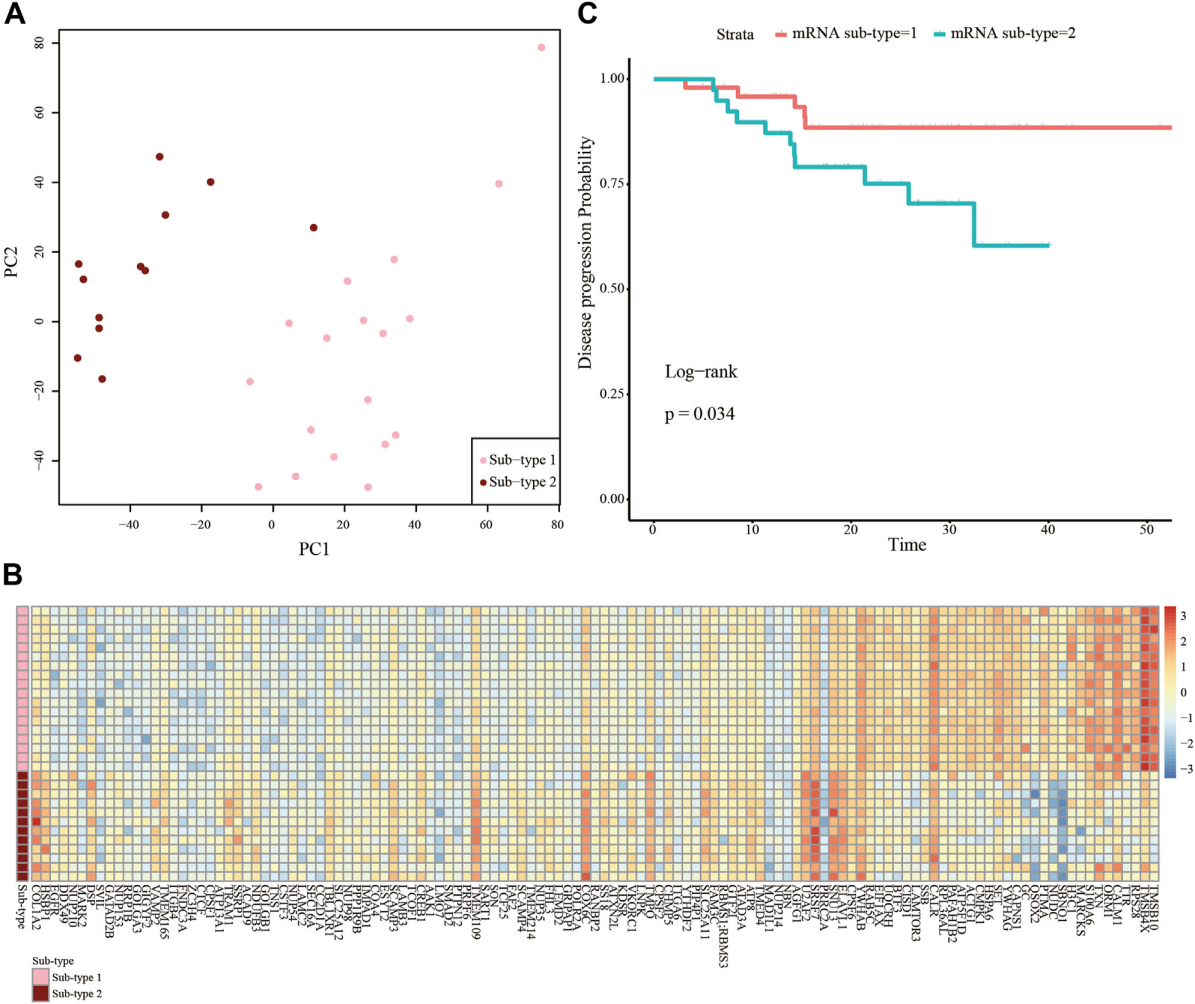
**The subtypes of NPC samples discovered by proteomics profiles.***A*, PCA analysis shows the separation of two NPC subtypes. *B*, heatmap for differentially expressed proteins between NPC subtypes. *C*, survival analysis using subtype-specific molecules and independent mRNA expression data. NPC, nasopharyngeal carcinoma; PCA, principal components analysis.

Using consensus clustering, we identified two NPC proteomics subtypes (see details in the Methods section and supplemental Fig. S5). Principal component analysis showed a clear separation between these two subtypes of NPC samples (Fig. 6*A*). Among the 30 NPC samples in the work, 18 belonged to subtype 1, and the other 12 samples were identified as subtype 2. There is no significant difference between the patients of these two subtypes in gender (*p*-value = 0.56, Fisher's Exact Test) or age (*p*-value = 0.95, ANOVA). To reveal subtype-specific molecular features, the differentially expressed proteins between the two subtypes of NPC were identified using the R package *limma*. With a cutoff of FDR <0.001, 125 proteins with differential expression between the two subtypes of NPC were found (Fig. 6*B*). These proteins were enriched in miRNA-related biological processes and ECM-receptor interaction pathways (supplemental Table S8). To verify whether these molecules could be subtype-specific features of NPC, we extracted the expression values of the mRNAs corresponding to these proteins from an independent study (31). The extracted mRNA expression data were used for consensus clustering. It was found that the NPC samples in this study could also be clustered into two groups (67 and 46 samples, supplemental Fig. S6). Furthermore, it was observed that these two subtypes obtained from the mRNA expression data had significantly different progression-free survival (Fig. 6*C*). When we analyzed the differentially expressed genes between the two subtypes in the mRNA expression data, we found that there were 24 genes differentially expressed between subtypes both at protein level in our dataset and at mRNA level in the public dataset (supplemental Table S9). Thus, the molecules identified in our proteomics data might be recognized as NPC subtype-specific features.

## Discussion

Since the advent of high-throughput techniques, especially large-scale whole-genome sequencing and RNA sequencing, the molecular signatures of NPC, including genomic alterations, susceptibility genes, gene expression patterns (both in host and EBV), host–virus interactions, and transcriptional features, have already been unveiled (44, 45, 46). However, proteins, as functional executors that are directly involved in all biological processes, can provide more valuable information and insights for understanding the disease, which may not be deciphered by genomic analysis alone. To date, there have been no large-scale proteomics studies to describe the proteome map of NPC. In this study, we identified a total of 7243 proteins with a 1% FDR from 30 primary NPC samples and 22 normal nasopharyngeal epithelial tissues through a normative proteomics procedure. Among these proteins, over 90% were present in both NPC and nasopharyngeal epithelial tissues. These data provide a proteome reference map of the human nasopharynx. Meanwhile, the differential proteins between NPC and control samples provided new insight into the molecular alterations and pathological processes of NPC and gave cues about therapeutic targets. Due to the special physiological structure and location of the nasopharynx, we could not obtain paired tumor-adjacent tissues of NPC like other solid tumors. The nasopharyngeal epithelial tissues used in this study were collected from patients diagnosed with nasopharyngeal inflammation. However, the proteome of these nasopharyngeal epithelial tissues may reflect the true protein composition of the human nasopharynx in a physiological state.

In addition to the expression alteration of proteins, the altered protein co-expression pattern may play a vital role in tumor progression owing to its reflection on direct protein interactions or indirect protein association. The evaluation of co-expression patterns underlies the importance of some specific proteins that are more prominent than others due to their centrality properties. Any changes in these proteins may lead to an abnormal connection between proteins and consequently result in a disease state. By exploring the differential protein co-expression patterns, we found that EGFR and SRC have an intense positive correlation in NPC samples compared to control samples. In fact, evidence has established that lapatinib, a dual inhibitor of EGFR and erb-b2 receptor tyrosine kinase 2, has a synergistic effect in NPC cells with an eEF-2 inhibitor by downregulating the SRC signaling pathway (47). These results suggest that inhibition of both EGFR and SRC may be an effective strategy in the treatment of NPC. In the KEGG pathway enrichment of differentially co-expressed protein pairs, pentose phosphate, and glycolysis gluconeogenesis pathways were identified as the most significant pathways. In addition, GPI, an important glycolytic enzyme, was identified as the most significantly differentially co-expressed link. These results all suggest that as the most remarkable features of cancer, glycolysis, and metabolic reprogramming are worthy of in-depth study in NPC. The integrative analysis of differential protein expression and differential co-expression helped to construct a unified molecular network for NPC. Compared to the differential expression analysis or differential co-expression analysis alone, the unified network revealed a more comprehensive proteomics landscape of NPC. For example, the unified network could enrich more hallmark gene sets that play important roles in carcinogenesis. In addition, the unified protein network suggested 15 druggable target candidates for NPC. Some of these druggable target candidates, such as HSP90 and CDK1, have been reported to be closely related to NPC progression (48, 49). HSP90, a well-known molecular chaperone, was demonstrated to be highly expressed in NPC and to be associated with poor prognosis of NPC (50). In this study, 17-AAG, a specific inhibitor of HSP90, was proven to significantly suppress the proliferation and promote the apoptosis of NPC cells and might be a potential therapeutic drug for NPC.

At present, the WHO subtype classification based on histopathology is still the most widely used form for NPC. However, an increasing number of clinicians realize that the current WHO classification is insufficient in NPC clinical diagnosis and treatment, as it cannot predict chemotherapy and radiotherapy outcomes or prognosis. Therefore, an increasing number of researchers have attempted to explore new classification systems for NPC from different perspectives. An integrative pharmacogenomics study revealed the molecular landscape and subtype-specific therapeutic response of NPC (51). In this study, we identified two NPC proteomics subtypes through consensus clustering and revealed the specific molecular features of the two subtypes. The subtype-specific molecular features were further verified by the corresponding mRNA expression value of those proteins, and distinctly different progression-free survival rates existed between the two subtypes. Unfortunately, we did not obtain the survival and prognosis information of the specimens used in this study and could not analyze the survival difference directly of the two subtypes. Further studies are planned that will include larger subject cohorts with survival information to verify the proteomics-based subtype classification established in this study.

Increasing research has demonstrated that integrated analyses of genomics, transcriptomics, proteomics, phosphoproteomics, and metabolomics would provide a complementary and more comprehensive understanding of tumors and identify new therapeutic targets (52, 53). However, integrative multiomics analysis based on the same specimen is impossible for NPC research owing to the undersized specimen. However, this situation will be improved with the widespread application of single-cell transcriptomics and single-cell proteomics. Meanwhile, the application of other models, such as patient-derived xenografts and patient-derived organoids, will be conducive to multiomics analysis for NPC (54).

In conclusion, we deciphered the proteomics landscape of human NPC and the nasopharynx and constructed a unified molecular network for NPC-based integrative analysis of differential protein expression and differential co-expression. The proteomics-based analysis identified two NPC subtypes with specific molecular features and different progression-free survival. It is expected that proteomics-driven precision medicine will bring good news to NPC patients in the future.

## Data availability

All MS proteomics raw data have been deposited to the ProteomeXchange Consortium *via* the iProX partner repository (access No. IPX0001265000, https://www.iprox.cn/page/home.html).

## Supplemental data

This article contains supplemental data.

## Conflict of interest

The authors declare no competing interests.

## Supplementary Data

Supplemental Table S9

Supplemental Figure S1 The distributions of iBAQ values of proteins in each samples described with boxplots. iBAQ, intensity-based absolute quantification.

Supplemental Figure S2 The distribution of the overall identified proteins at diverse cell locations.

Supplemental Figure S3 Top two subnetworks most significantly enriched with differential co-expression links. The overlapped nodes and edges between these two subnetworks are in red.

Supplemental Figure S4 The estimated activity of Epstein-Barr virus infection pathway was significantly correlated with IFN-gamma response.

Supplemental Figure S5 Consensus clustering results produced by R package *ConsensusClusterPlus* with different cluster numbers k using proteomics data.

Supplemental Figure S6 Consensus clustering results produced by R package *ConsensusClusterPlus* with different cluster numbers k using mRNA expression data.
