# Supplementary figures and images for: Proteomics Profiling Reveals the Molecular Signatures and Potential Therapeutic Targets of Human Nasopharyngeal Carcinoma
Source: Mol Cell Proteomics. 2023 May 11;22(6):100567. doi: 10.1016/j.mcpro.2023.100567 (PMC10326745; doi:10.1016/j.mcpro.2023.100567)

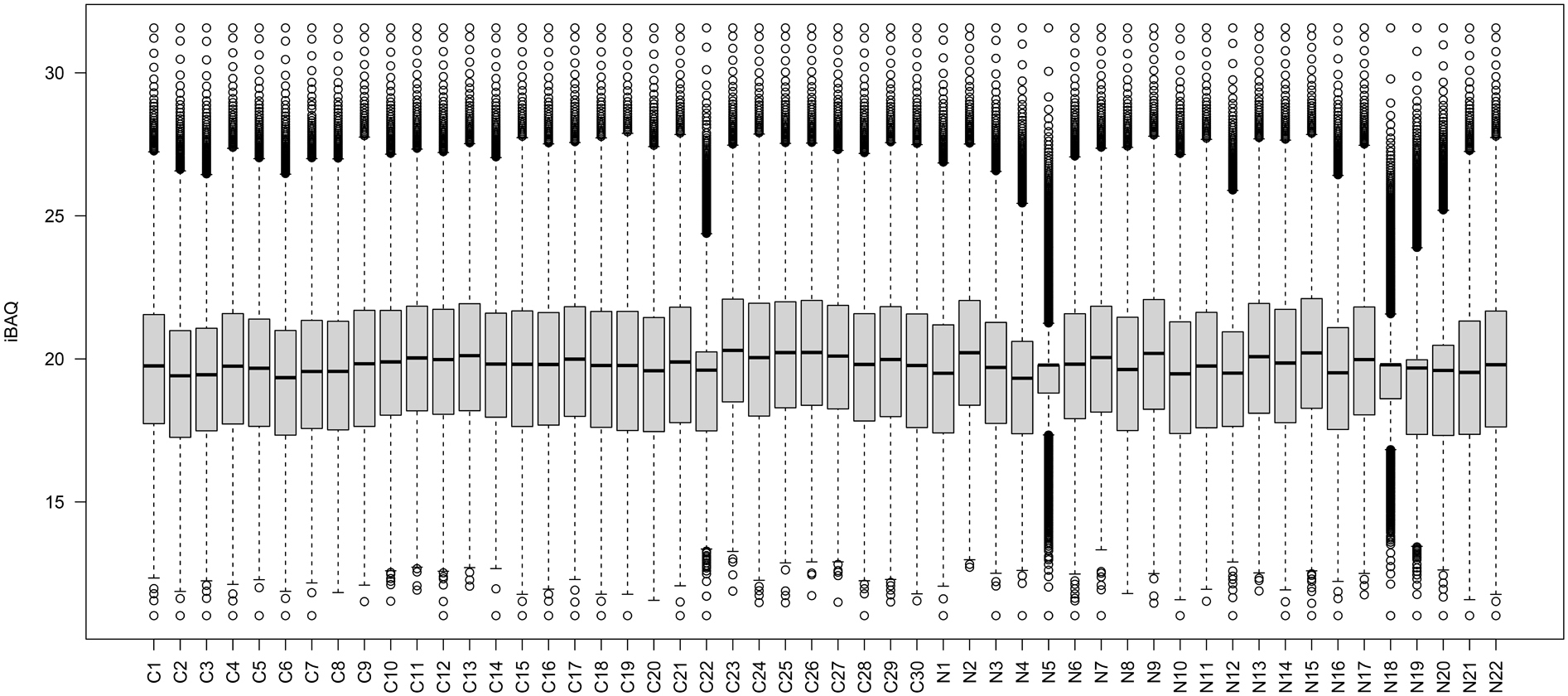

Supplement: Supplemental Figure S1 — The distributions of iBAQ values of proteins in each samples described with boxplots. iBAQ, intensity-based absolute quantification. [file figs1.jpg]

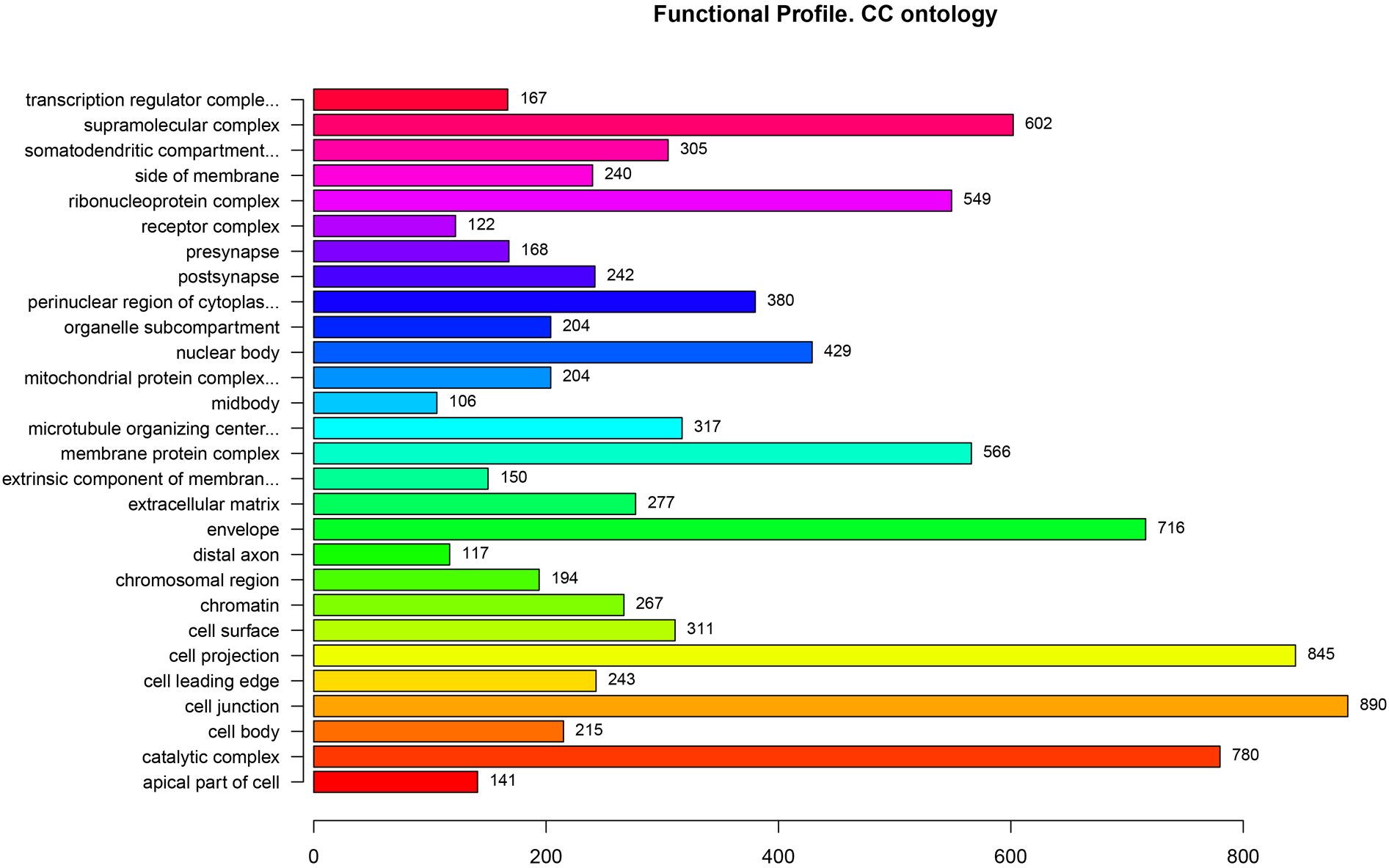

Supplement: Supplemental Figure S2 — The distribution of the overall identified proteins at diverse cell locations. [file figs2.jpg]

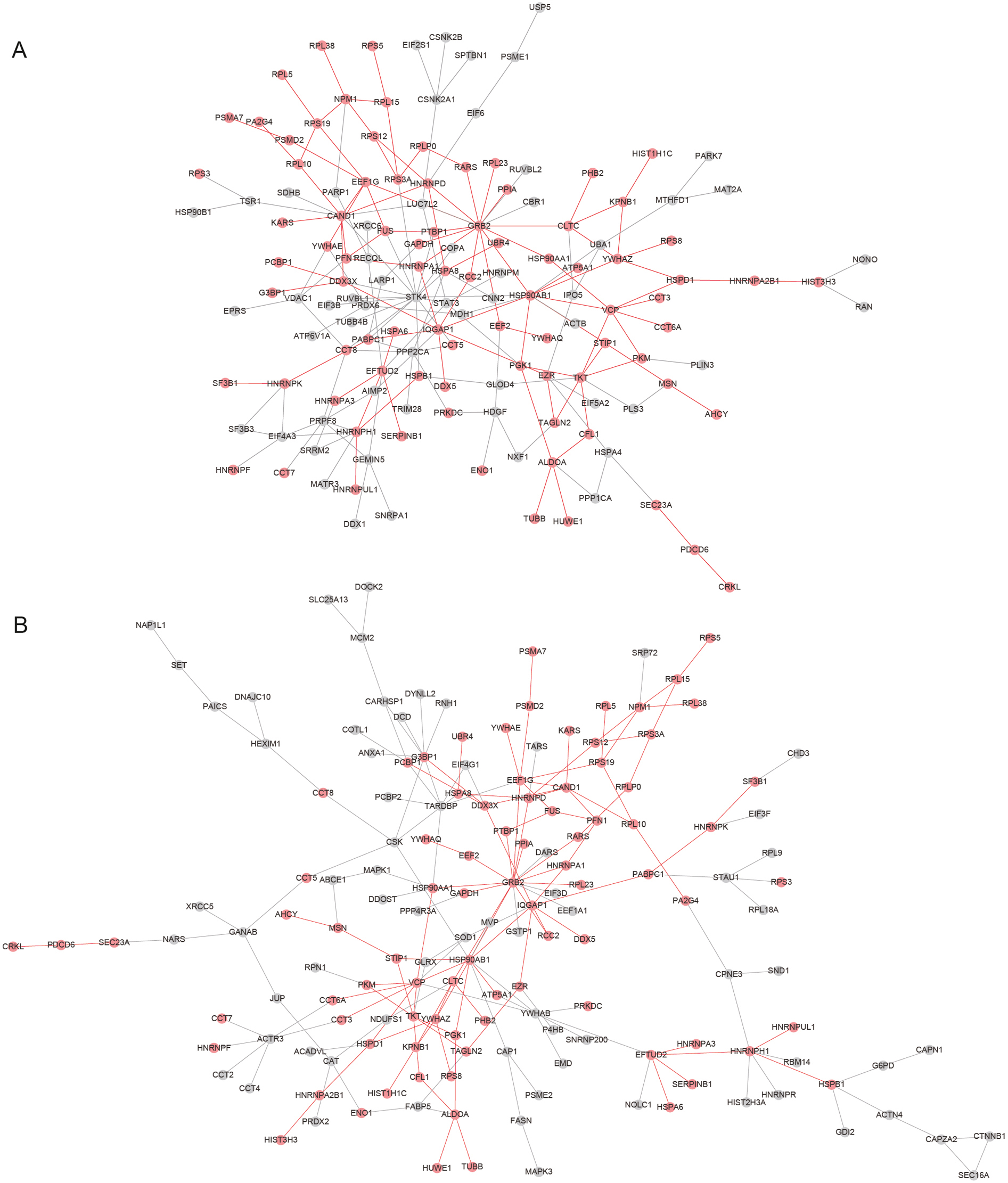

Supplement: Supplemental Figure S3 — Top two subnetworks most significantly enriched with differential co-expression links. The overlapped nodes and edges between these two subnetworks are in red. [file figs3.jpg]

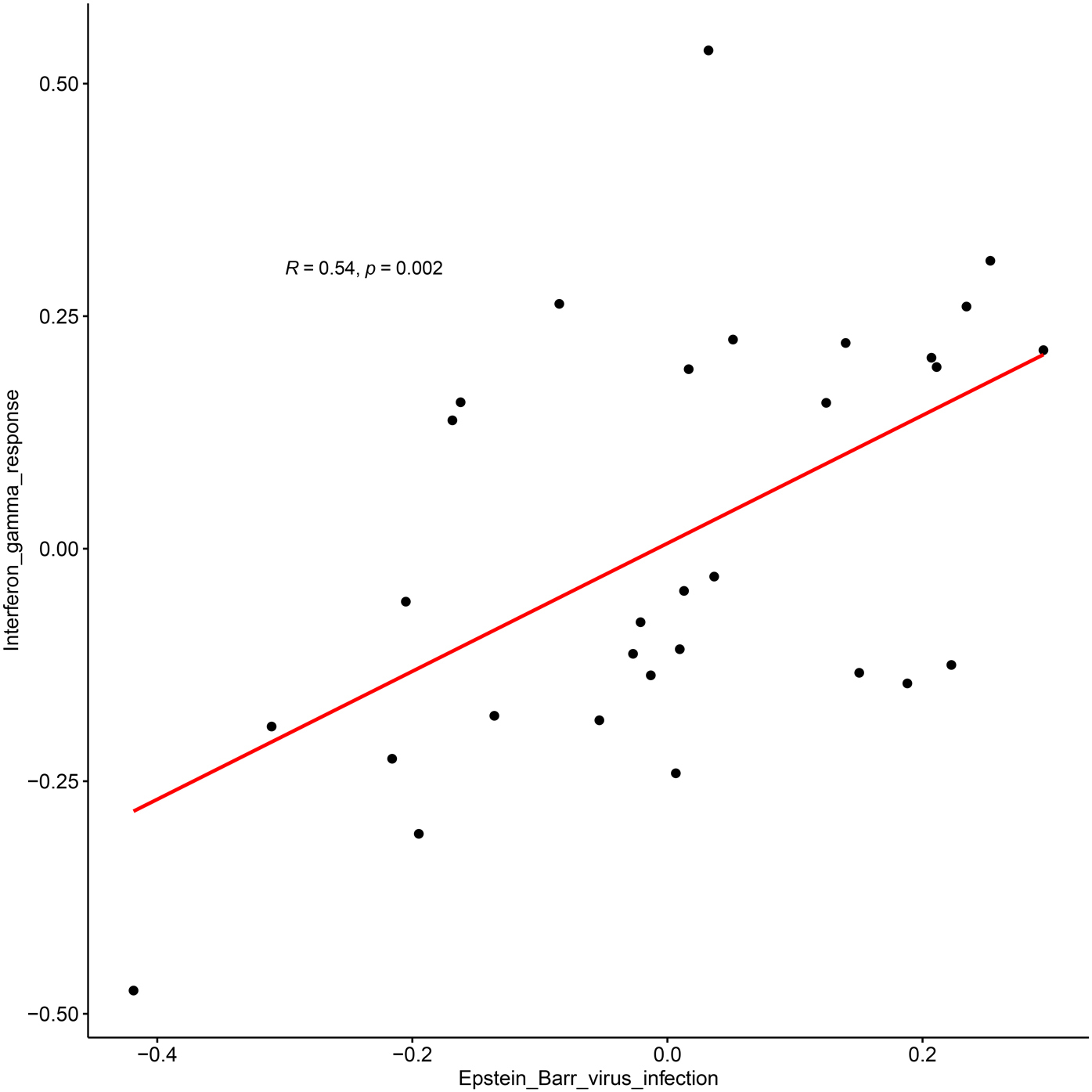

Supplement: Supplemental Figure S4 — The estimated activity of Epstein-Barr virus infection pathway was significantly correlated with IFN-gamma response. [file figs4.jpg]

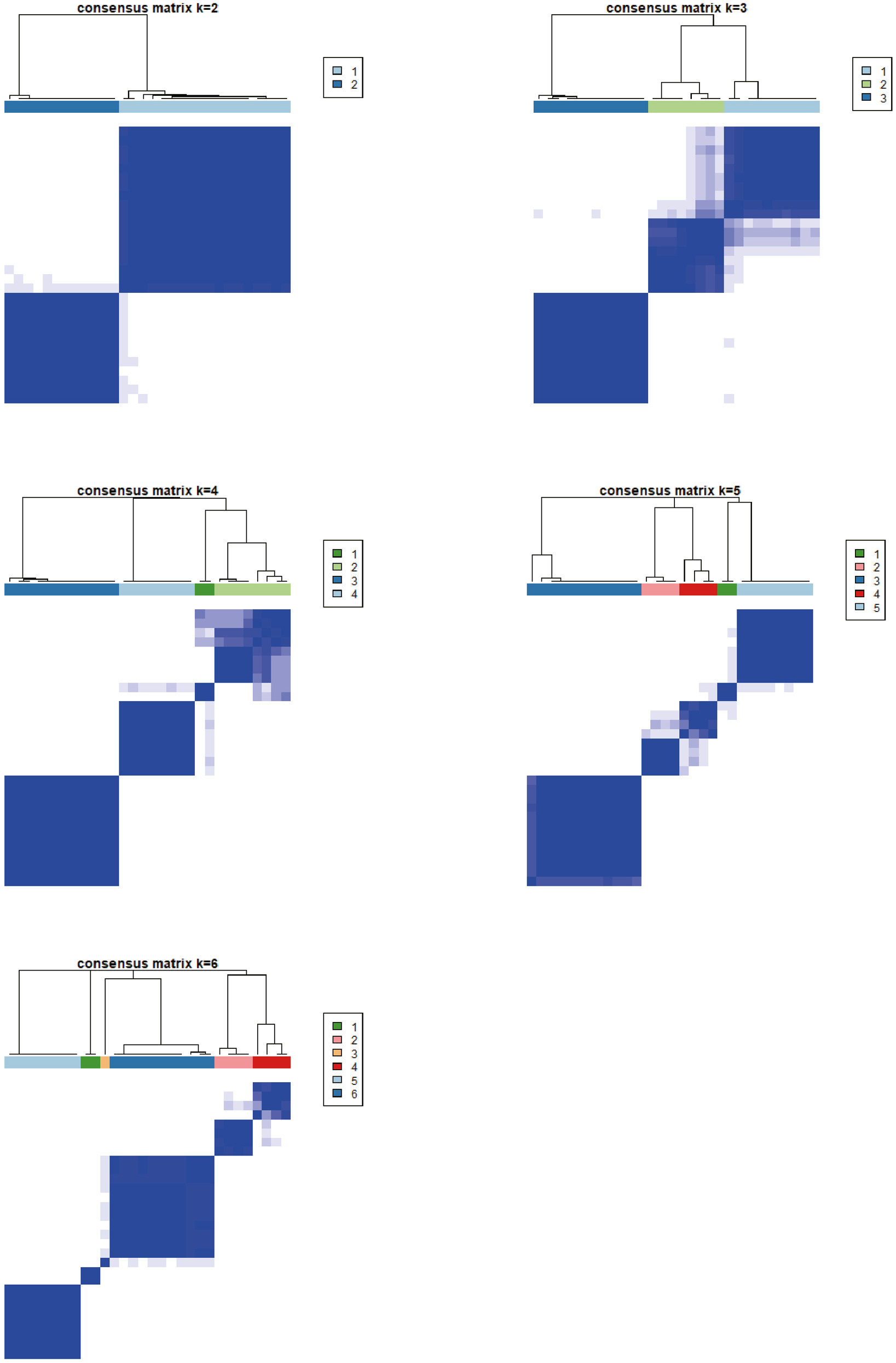

Supplement: Supplemental Figure S5 — Consensus clustering results produced by R package ConsensusClusterPlus with different cluster numbers k using proteomics data. [file figs5.jpg]

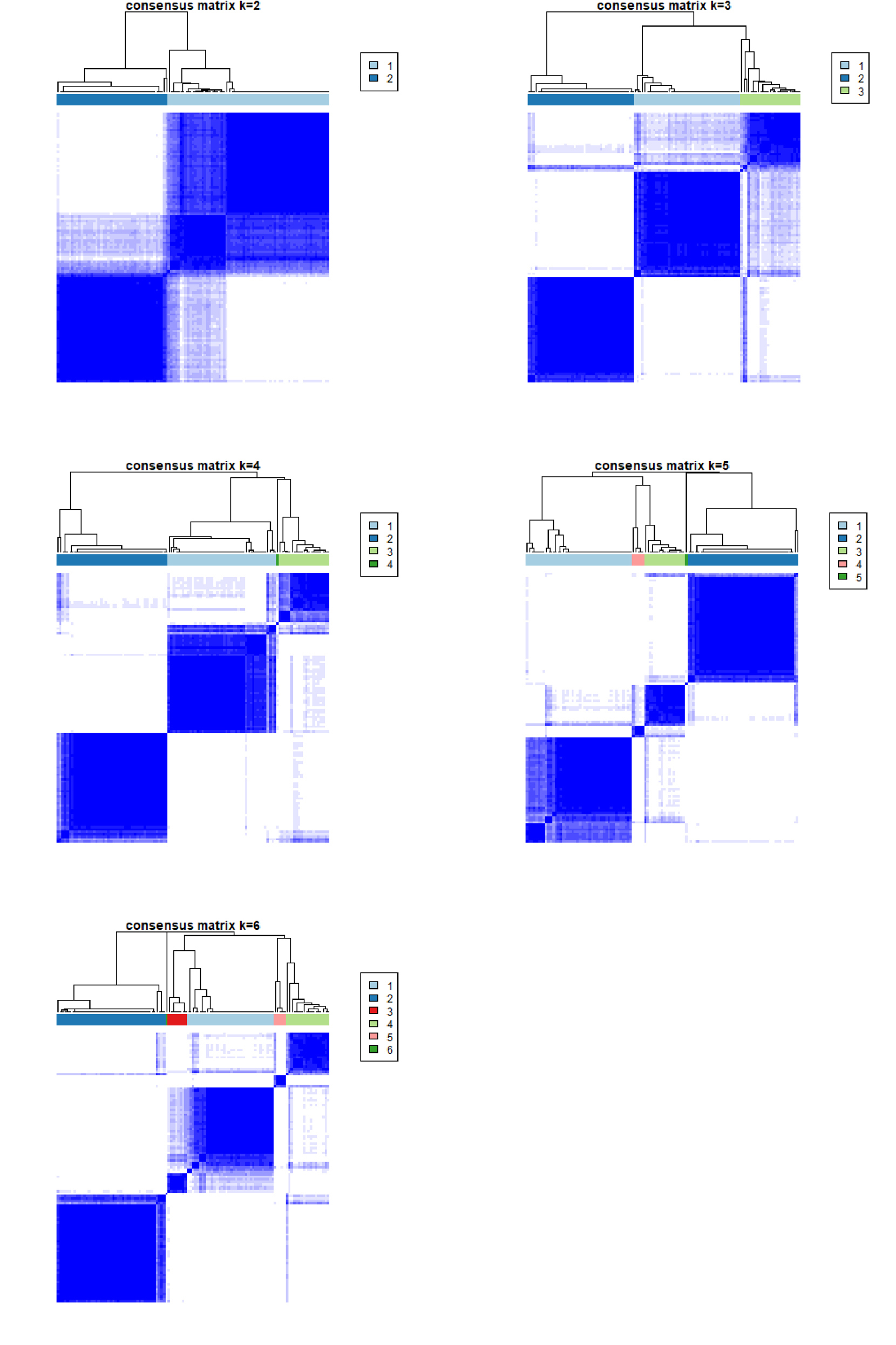

Supplement: Supplemental Figure S6 — Consensus clustering results produced by R package ConsensusClusterPlus with different cluster numbers k using mRNA expression data. [file figs6.jpg]
